# Factors influencing knee valgus alignment in Crowe type IV hip dysplasia after total hip arthroplasty

**DOI:** 10.1186/s10195-021-00601-y

**Published:** 2021-10-16

**Authors:** Jing-yang Sun, Hai-yang Ma, Jun-min Shen, Yin-qiao Du, Yu Dong, Yan-chao Zhang, Yong-gang Zhou, Yan Wang

**Affiliations:** 1 Medical School of Chinese PLA, Beijing, 100853 China; 2 Senior Department of Orthopedics, The Fourth Medical Center of PLA General Hospital, National Clinical Research Center for Orthopedics, Sports Medicine & Rehabilitation, Fuxing Road, Haidian District, Beijing, 100853 China

**Keywords:** Hip dysplasia, Crowe type IV, Knee valgus alignment, Total hip arthroplasty

## Abstract

**Background:**

Valgus deformity of the knee remains a complaint after total hip arthroplasty (THA) among some patients with Crowe type IV hip dysplasia. We aimed to identify the knee alignment in these patients before and after surgery, and to explore the factors contributing to postoperative knee valgus alignment.

**Materials and methods:**

We retrospectively reviewed a series of Crowe type IV patients who received THA between February 2010 and May 2019 in our hospital. The patients’ medical data were collected from the hospital information system. On both preoperative and postoperative full limb length standing radiographs, the following parameters were measured: hip–knee–ankle angle (HKA), mechanical lateral distal femoral angle (mLDFA), medial proximal tibial angle, anatomical tibiofemoral angle, anatomical lateral distal femoral angle, femoral neck-shaft angle, pelvic obliquity, limb length, height and lateral distance of hip center, and femoral offset. Univariate and multivariate binary logistic regression were used to identify the factors influencing postoperative knee valgus alignment.

**Results:**

A total of 64 Crowe type IV patients (87 hips) were included in the study. Overall, HKA improved from 176.54 ± 3.52° preoperatively to 179.45 ± 4.31° at the last follow-up. Those hips were subdivided into non-valgus group (≥ 177.0°, *n* = 65) and valgus group (< 177.0°, *n* = 22) according to postoperative HKA. Only postoperative mLDFA was a significant factor in the multivariate regression model.

**Conclusions:**

The postoperative mLDFA is a major factor related to knee valgus alignment after THA, which combines the preoperative anatomy and surgical reconstruction. Other factors previously published were found to have no significance.

**Level of evidence:**

III.

## Introduction

Total hip arthroplasty (THA) for patients with Crowe type IV dysplasia is a challenging surgical procedure [1]. Although recent clinical reports have shown excellent outcomes in these hips, valgus deformity of the knee remains a complaint after surgery among some patients [2, 3].

As observed in previous studies, valgus deformity of the knee can be explained by some developmental changes in the osseous anatomy, including increase in medial femoral condyle height, decrease in lateral femoral condyle height, and higher medial proximal tibial angle [4–7]. Furthermore, Kilicarslan et al. reported that adaptive changes occur postoperatively in ipsilateral knee joints that were normal before THA in Crowe type III–IV hips. When the cup component was implanted at the true acetabulum, the limb was substantially lengthened, resulting in stiffness and tightness of the iliotibial tract. They hypothesized that tension along the iliotibial tract might force the knee into valgus deformity [3, 8]. Except for considerations of structural alterations, Kandemir et al. thought knee valgus can also develop because of adaptive posture when walking [4]. They speculated that medialization of the hip also led to medialization of the knee. Under this circumstance, the patient would try to walk either with the knees closer together to keep the joint line horizontal, or with wider interfoot distance to avoid striking the contralateral knee. Both of these postures may predispose the knee to valgus deformity. Therefore, they suggested using a high-offset femoral component to compensate for the medialization of the hip center.

Until now, although the natural history of knee joint among Crowe type IV patients is well defined in the literature, there have not been many studies of the changes of knee alignment after THA [4–6, 9–11]. Kocabiyik et al. found that, in a cohort of 25 Crowe type IV patients treated with THA followed for 1 year, hip–knee–ankle (HKA) angle significantly improved from −1.6 ± 6.1° preoperatively to 1.7 ± 7.2° postoperatively. They supposed that modification of femoral offset and reconstruction of hip center led to neutralization of knee valgus alignment [12]. Moreover, in a retrospective study of 50 unilateral Crowe type IV patients treated with THA, Zhao et al. found that HKA angle was significantly larger immediately after surgery than before (175.36 ± 2.67° versus 177.25 ± 2.09°), and almost unchanged at 2-year follow-up [13]. Indeed improvement of knee alignment benefited by surgery has been observed according to the average radiographic parameters. However, there has been no study focusing on the actual status of their knee alignment after surgery and the factors influencing postoperative knee alignment.

Therefore, the aim of this study was to identify the knee alignment in patients with Crowe type IV hip dysplasia both preoperatively and postoperatively, and to explore the factors contributing to postoperative knee valgus alignment.

## Materials and methods

### Patients

We retrospectively reviewed a series of Crowe type IV patients who received THA between February 2010 and May 2019 in our hospital by a single surgeon. The inclusion criteria were as follows: patients with Crowe type IV hip dysplasia on at least one side, patients who had a full limb length standing anteroposterior (AP) radiograph both preoperatively and at least 1 year postoperatively, patients were followed for a minimum of 1 year, and patients whose profile of the limb were recorded at the outpatient follow-up. Exclusion criteria were as follows: patients with an angulation or arcuate deformity at the diaphysis of femur or tibia, patients with advanced osteoarthritis (Kellgren–Lawrence grade III–IV) of either knee, patients with neuromuscular disease, and nonstandard radiographs that cannot be measured because of improper flexion or rotation of the knee. The patients’ medical data were collected from the hospital information system. Before data collection, an institutional review board approved the study design and all patients consented to allow analysis of their data.

### Surgical procedure

All operations were performed by one senior surgeon under general anesthesia, through posterolateral approach in the lateral decubitus position. The cup was implanted at the anatomic position. No structural or morselized autograft were used. After the cup implantation, the femoral canal was prepared using the dedicated reamer for the S-ROM stem (DePuy, Warsaw, Indiana). If hip reduction with a femoral trial stem was difficult, a subtrochanteric osteotomy would be performed for femoral shortening. The osteotomy position was planned to be 1–2 cm beneath the lesser trochanter. After trial reduction, stability, limb length, and soft tissue tension were evaluated. Limb length discrepancy (LLD) was assessed according to relative position of the inferior point of bilateral patella. Mild LLD could be adjusted by adjusting head/neck length and stem depth in femur. Finally, the definitive femoral component was implanted with the version to allow approximately 30–50° of combined anteversion. At the end of the surgery, hip abduction was tested to evaluate the necessity of a percutaneous partial adductor tenotomy. Postoperatively, both the hip and knee joint were maintained in flexion for several days to relax the sciatic nerve and reduce tension of soft tissue.

The clinical outcome was assessed by the Harris Hip Score (HHS). Perioperative complications such as dislocation, fracture, infection, and nerve injury were recorded.

### Radiographic measurement

All full limb length standing AP radiographs were obtained using a standard protocol by GE Definium 6000 digital radiography (DR) system (GE Healthcare, USA) [14]. Patients were required to face the X-ray tube in a standing position with the tibial tubercle pointing anteriorly. Patients were asked to keep their legs straight and allowed tiptoeing, without the need to place wooden blocks under the short limb to make the pelvis level. The radiographs were viewed and measured on the PACS software in hospital (Medcare, Qingdao, China). On both preoperative and postoperative radiographs, the following parameters were measured in each affected limb:

The hip–knee–ankle angle (HKA): the lateral angle between the mechanical axis of femur and tibia. The lower limb alignment was defined as neutral when the HKA was between 177.0° and 183.0°, valgus when the HKA was < 177.0°, and varus when the HKA was > 183.0°.

The mechanical lateral distal femoral angle (mLDFA): the lateral angle between the joint line of distal femur and the femoral mechanical axis. The normal value of mLDFA was between 85° and 90°. Value > 90.0° was defined to contribute to varus alignment. Value < 85.0° was defined to contribute to valgus alignment.

The mechanical medial proximal tibial angle (mMPTA): the medial angle between the joint line of tibial plateau and the tibial mechanical axis (normal value 85–90°). Value > 90.0° was defined to contribute to valgus alignment. Value < 85.0° was defined to contribute to varus alignment.

The anatomical tibiofemoral angle (aTFA): the lateral angle between the anatomical axis of femur and tibia (normal value 170.0–175.0°). The lower limb alignment was defined as valgus when the aTFA was < 170.0°, and varus when the aTFA was > 175.0°.

The anatomical lateral distal femoral angle (aLDFA): the lateral angle between the joint line of distal femur and the femoral anatomical axis (normal value 79–83°). The femoral anatomical axis was described as the line through the center of the femoral medullary canal. Value > 83.0° was defined to contribute to varus alignment. Value < 79.0° was defined to contribute to valgus alignment.

The femoral neck-shaft angle (NSA): the medial angle between the femoral neck axis and the femoral shaft axis.

The pelvic obliquity: the angle between the inter-teardrop line and the horizontal line. A positive value meant the pelvis leaning with the ipsilateral side downward. For bilateral cases, the value of one side is negative to the other.

The limb length: the distance from the base of teardrop to the prominence of medial malleolus. The teardrop was identified by reference to preoperative radiograph when it was violated due to medialization of the cup component. LLD was the length difference of both lower limbs. A positive LLD value meant a longer limb length than that of contralateral side. For bilateral cases, the value of one side is negative to the other.

The height of hip center (HHC): the perpendicular distance from the center of femoral head to the inter-teardrop line.

The lateral distance of hip center (LHC): the horizontal distance from the center of femoral head to the lateral border of ipsilateral teardrop, which was parallel to the inter-teardrop line.

The femoral offset (FO): the perpendicular distance from the center of femoral head to the axis of femoral medullary canal.

The measurement techniques and scale of radiographic parameters were by reference to previous literature [5, 6, 11, 15–17]. Limb lengthening, ΔHHC, ΔLHC, and ΔFO were calculated as the postoperative value minus the preoperative value. Before embarking on the study, all observers reached an agreement on criteria for radiographic measurement and all identifying masks were removed. Measurement was performed twice by two observers independently in random order, with an interval of at least 1 month. Interobserver variability was measured by comparing the average value of two observers, while intraobserver reliability was determined by comparing the two measurements of each observer. Assessment of inter- and intraobserver consistency was accomplished by the use of the intraclass correlation coefficient (ICC). Agreement was graded as slight (0–0.2), fair (0.21–0.40), moderate (0.41–0.60), substantial (0.61–0.80) or almost perfect (0.81–1.0) [18].

### Statistical analyses

All statistical analyses were performed using SPSS version 26.0 (IBM Inc., Armonk, New York). *p* < 0.05 was considered statistically significant. Categorical variables were presented as frequencies, and continuous variables as means and standard deviation. Categorical variables were compared using chi-square test. A paired t-test was used to compare the preoperative and postoperative radiographic parameters. Binary logistic regression was used to identify the preoperative and postoperative anatomical factors, and surgical factors contributing to postoperative knee valgus alignment. Odds ratios (ORs) and 95% confidence intervals (CIs) were calculated for these results. The factors whose *p*-value was less than 0.1 and 95% CI of OR did not contain 1 were then included in a multivariate analysis using a binary logistic algorithm. Multicollinearity of the variables was assessed by collinearity check using variable inflection factors (VIF < 5).

## Results

A total of 64 Crowe type IV patients (87 hips) were included in the study. There were 4 males and 60 females. The average age was 39.7 ± 10.9 years (range 19–64 years). The average duration of follow-up was 23.7 ± 21.0 months (range 12–108 months). Of these patients, 41 had unilateral hip dislocation and 23 had bilateral dislocation. Regarding the radiographic measurements, the intra- and interobserver agreements showed nearly perfect reliability (ICC > 0.81). Overall, HKA improved from 176.54 ± 3.52° (range 166.42–183.44°) preoperatively to 179.45 ± 4.31° (range 168.81–191.96°) at the last follow-up.

**Fig. 1:**
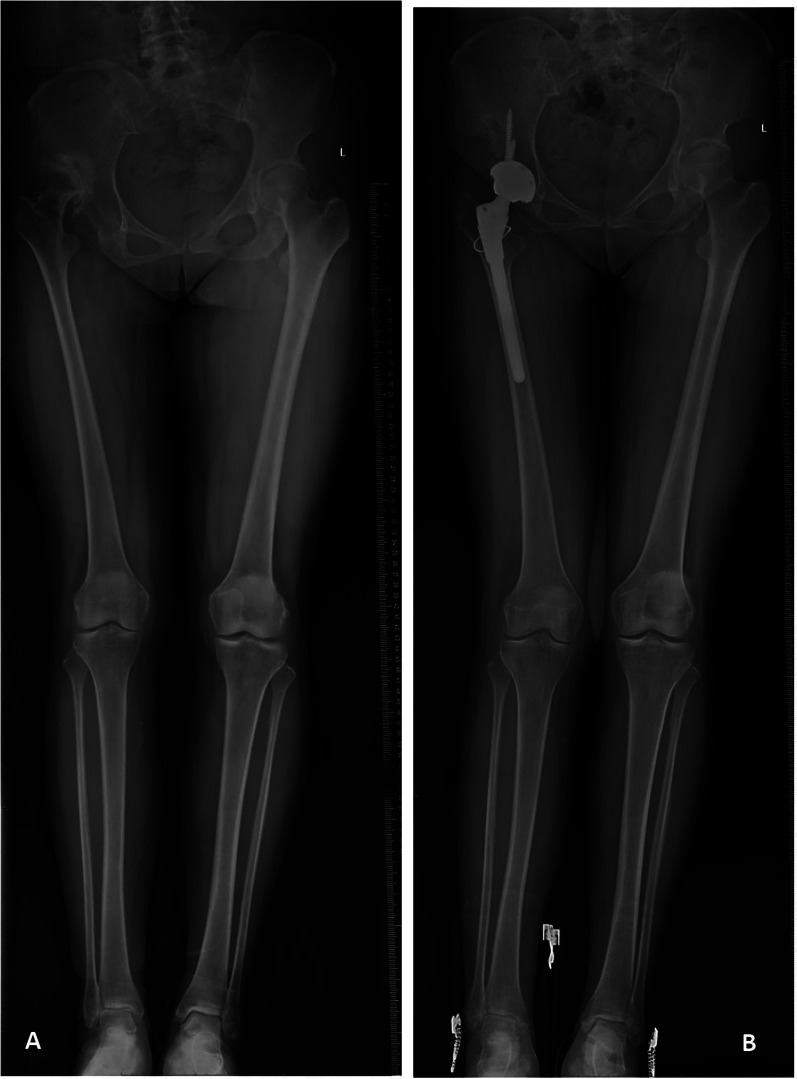
Valgus alignment of the knee in a 54-year-old Crowe type IV patient after total hip arthroplasty. **A** Preoperative radiograph. **B** Postoperative radiograph

**Fig. 2:**
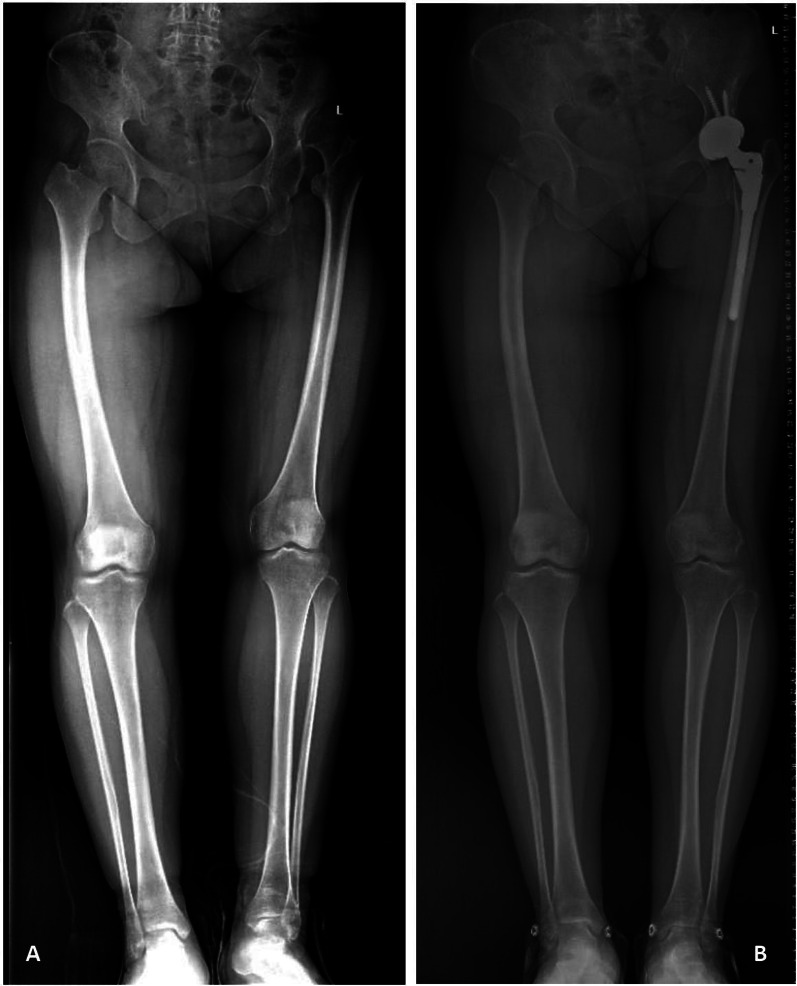
Non-valgus alignment of the knee in a 50-year-old Crowe type IV patient after total hip arthroplasty. **A** Preoperative radiograph. **B** Postoperative radiograph

Based on postoperative HKA, those hips were subdivided into non-valgus group (≥ 177.0°, *n* = 65) and valgus group (< 177.0°, *n* = 22) (Figs. 1 and 2). In the non-valgus group, HHS improved from 39.05 ± 4.68 to 88.49 ± 4.66 after THA. While in the valgus group, it improved from 38.27 ± 4.05 to 85.09 ± 3.95. The difference of postoperative HHS between these two groups was of statistical significance (*p* = 0.003). Two postoperative dislocations occurred. One happened owing to a fall during the intermittent period of staged bilateral THA, and was treated by open reduction. The other happened during early squatting exercise, and was treated by closed reduction under anesthesia. At the last follow-up, no patients complained of muscular tension on the lateral side of their knees in either group.

The change of anatomical factors after surgery in both the non-valgus and valgus groups is presented in Table 1. Significant differences before and after surgery were observed in LLD, HHC, LHC, and FO in both non-valgus and valgus groups. Neither group showed a significant change in NSA. Moreover, unlike the non-valgus group, no evident change of HKA, mLDFA, aTFA, and pelvic obliquity was seen in the valgus group.

**Table 1:** Comparison of anatomical factors before and after surgery in both non-valgus and valgus groups

		Non-valgus group			Valgus group	
Variable	Before surgery	After surgery	*p* value	Before surgery	After surgery	*p* value
HKA (°)	177.66 ± 2.96	181.16 ± 3.30	< 0.001	173.15 ± 2.88	174.26 ± 2.51	0.096
mLDFA (°)	83.83 ± 3.11	86.98 ± 3.28	< 0.001	81.50 ± 2.29	82.50 ± 2.20	0.138
aTFA (°)	172.94 ± 11.82	176.45 ± 3.22	0.02	164.87 ± 19.94	170.20 ± 3.53	0.213
LLD (mm)	−11.93 ± 20.16	−0.69 ± 10.80	< 0.001	−21.20 ± 19.05	−4.65 ± 11.25	0.001
HHC (mm)	74.42 ± 18.76	18.61 ± 5.13	< 0.001	71.74 ± 25.06	16.76 ± 7.84	< 0.001
LHC (mm)	49.10 ± 11.45	23.61 ± 3.66	< 0.001	47.80 ± 18.22	22.82 ± 4.97	< 0.001
FO (mm)	23.81 ± 9.28	33.51 ± 4.81	< 0.001	25.03 ± 10.92	31.13 ± 4.78	0.028
NSA (°)	137.09 ± 16.58	137.60 ± 4.43	0.818	133.58 ± 15.18	137.89 ± 5.52	0.206
Pelvic obliquity (°)	3.12 ± 6.86	0.07 ± 5.34	< 0.001	5.70 ± 7.29	3.02 ± 5.73	0.069

*HKA* hip–knee–ankle angle, *mLDFA* mechanical lateral distal femoral angle, *aTFA* anatomical tibiofemoral angle, *LLD* limb length discrepancy, *HHC* height of hip center, *LHC* lateral distance of hip center, *FO* femoral offset, *NSA* neck-shaft angle

The univariate analysis of demographics and preoperative anatomical factors showed that the preoperative HKA, mLDFA, and aLDFA were related to the knee valgus alignment after surgery. Higher value of these angles was associated with lower rates of knee valgus alignment (Table 2). Among surgical and postoperative anatomical factors, the postoperative mLDFA, aTFA, and pelvic obliquity were determined to be the influencing factors on knee valgus alignment after surgery. Increased mLDFA and aTFA, and decreased pelvic obliquity after surgery were associated with lower rates of knee valgus alignment (Table 3).

**Table 2:** Univariate analysis of demographics and preoperative anatomical factors

Variable	Non-valgus group (65 hips)	Valgus group (22 hips)	OR (95% CI)	*p* value
Mean age (years)	37.7 ± 11.3	46.9 ± 7.5	1.109 (0.975–1.261)	0.117
Sex, M:F	4:61	1:21	0.726 (0.077–6.867)	0.780
Mean height (m)	1.59 ± 0.14	1.58 ± 0.05	0.639 (0.007–55.986)	0.639
Mean weight (kg)	54.5 ± 10.0	56.8 ± 7.5	1.026 (0.976–1.077)	0.318
Mean BMI (kg/m^2^)	21.69 ± 3.65	22.66 ± 2.92	1.085 (0.941–1.252)	0.262
Previous hip surgery, *n* (%)				0.739
Yes	8 (12.3)	4 (18.2)	Reference	
No	57 (87.7)	18 (81.8)	0.632 (0.170–2.345)	0.492
Affected sides, *n* (%)				0.420
Unilateral	29 (44.6)	12 (54.5)	Reference	
Bilateral	36 (55.4)	10 (45.5)	0.671 (0.254–1.773)	0.421
HKA (°)	177.66 ± 2.96	173.15 ± 2.88	0.602 (0.469–0.772)	< 0.001
mLDFA (°)	83.83 ± 3.11	81.50 ± 2.29	0.751 (0.614–0.918)	0.005
mMPTA (°)	88.13 ± 3.25	89.30 ± 3.31	1.118 (0.961–1.300)	0.150
aLDFA (°)	80.32 ± 3.03	77.53 ± 3.46	0.757 (0.633–0.905)	0.002
aTFA (°)	172.94 ± 11.82	164.87 ± 19.94	0.962 (0.915–1.012)	0.136
Pelvic obliquity (°)	3.12 ± 6.86	5.70 ± 7.29	1.056 (0.983–1.135)	0.139
LLD (mm)	− 11.93 ± 20.16	− 21.20 ± 19.05	0.992 (0.968–1.017)	0.072

*BMI* body mass index, *HKA* hip–knee–ankle angle, *mLDFA* mechanical lateral distal femoral angle, *mMPTA* mechanical medial proximal tibial angle, *aLDFA* anatomical lateral distal femoral angle, *aTFA* anatomical tibiofemoral angle, *LLD* limb length discrepancy, *CI* confidence interval, *OR* odds ratio

**Table 3:** Univariate analysis of surgical and postoperative anatomical factors

Variable	Non-valgus group (65 hips)	Valgus group (22 hips)	OR (95% CI)	*p* value
Femoral shortening osteotomy, *n* (%)				0.819
Yes	46 (70.8)	15 (68.2)	Reference	
No	19 (29.2)	7 (31.8)	1.130 (0.398–3.210)	0.819
Limb lengthening (mm)	31.41 ± 12.85	38.41 ± 16.06	1.037 (0.995–1.081)	0.082
mLDFA (°)	86.98 ± 3.28	82.50 ± 2.20	0.582 (0.451–0.750)	< 0.001
aTFA (°)	176.45 ± 3.22	170.20 ± 3.53	0.504 (0.372–0.684)	< 0.001
ΔHHC (mm)	55.81 ± 19.58	54.98 ± 26.18	0.998 (0.974–1.022)	0.879
ΔLHC (mm)	−25.49 ± 11.10	−24.99 ± 17.77	1.003 (0.964–1.044)	0.881
ΔFO (mm)	9.70 ± 9.79	6.10 ± 11.48	0.966 (0.919–1.016)	0.183
ΔNSA (°)	0.51 ± 17.08	4.32 ± 14.75	1.015 (0.983–1.047)	0.371
Pelvic obliquity (°)	0.07 ± 5.34	3.02 ± 5.73	1.111 (1.008–1.226)	0.035
LLD (mm)	−0.69 ± 10.80	−4.65 ± 11.25	0.966 (0.921–1.014)	0.161

*mLDFA* mechanical lateral distal femoral angle, *aTFA* anatomical tibiofemoral angle, *LLD* limb length discrepancy, *HHC* height of hip center, *LHC* lateral distance of hip center, *FO* femoral offset, *NSA* neck-shaft angle, *CI* confidence interval, *OR* odds ratio

The above six variables (preoperative HKA, mLDFA, and aLDFA, and postoperative mLDFA, aTFA, and pelvic obliquity) were included in the multivariate regression model. The results showed that postoperative mLDFA was the only significant factor in the adjusted model and the other factors were no longer considered as independent influencing factors on knee valgus alignment (Table 4). No problem of collinearity was found.

**Table 4:** Statistical results of multivariate model

Variable	OR (95% CI)	*p* value
Preoperative HKA	0.663 (0.406–1.084)	0.101
Preoperative mLDFA	1.116 (0.742–1.679)	0.599
Preoperative aLDFA	1.130 (0.840–1.520)	0.418
Postoperative mLDFA	0.586 (0.384–0.894)	0.013
Postoperative aTFA	0.705 (0.484–1.026)	0.068
Postoperative pelvic obliquity	1.064 (0.922–1.228)	0.394

*HKA* hip–knee–ankle angle, *mLDFA* mechanical lateral distal femoral angle, *aLDFA* anatomical lateral distal femoral angle, *aTFA* anatomical tibiofemoral angle, *CI* confidence interval, *OR* odds ratio

## Discussion

It is not rare to see knee valgus deformity in Crowe type IV hips after THA, which may be a potential complaint of those patients [3.13]. Current literature mainly focused on the knee joint among untreated Crowe type IV patients [4–6, 9–11]. To the best of our knowledge, this is the first study to report the factors contributing to postoperative knee valgus alignment. Based on the results of the multivariate regression model, we identified that postoperative mLDFA was a major factor, the increase of which would lower the rate of knee valgus alignment after surgery.

Both the reports of Kocabiyik et al. and Zhao et al. revealed that knee alignment was significantly improved after THA, according to the overall HKA angle [12, 13]. However, neither of these studies considered the actual status of postoperative knee alignment. Therefore, we subdivided the included hips into non-valgus and valgus groups based on postoperative HKA. In the comparison of anatomical factors before and after surgery, no significant change of HKA, mLDFA, aTFA, or pelvic obliquity was found in the valgus group. These results implied that not all knee alignments improved after THA.

As previous studies suggested, postoperative knee valgus deformity can be attributed to three factors, including intrinsic osseous dysplasia, iliotibial tract tension resulting from limb lengthening, and adaptive change of posture secondary to the hip reconstruction [3, 4, 11]. With regard to the osseous anatomy, developmental dysplasia at the proximal end, diaphysis, or distal end of femur or tibia can all effect HKA angle. Excluding the cases with angulation or bow deformity in the coronal plane, we depicted the deformity at different positions by radiographic parameters. Similar to previous studies, lower mLDFA and aLDFA, and higher mMPTA were observed before surgery, especially in the valgus group [4, 5]. However, mMPTA was not a significant factor based on univariate analysis, which implied that a valgus tibial plateau did not contribute much to postoperative knee valgus alignment. According to our univariate analysis results, preoperative HKA, mLDFA, and aLDFA, and postoperative aTFA were significantly associated with knee valgus alignment after THA. However, in the multivariate analysis, their significance was lost. This could be explained by the variations in NSA, femoral offset before surgery, and reconstruction of hip center on femoral side [19, 20]. In other words, the only significant factor, postoperative mLDFA, was the combination of intrinsic anatomical factors and surgical factors that worked together. Inadequate reconstruction of mLDFA was related to a higher rate of knee valgus alignment. Interestingly, there were no significant differences in the average change of hip center position, NSA, or femoral offset between two groups. In consideration of the larger standard deviation of those parameters, we speculated that it was a consequence of offset among different individual hip reconstructions.

Limb lengthening was inevitable when reducing the displaced hips into the anatomical hip center, which can lead to tension of neuromuscular structures [3, 8, 21]. Kilicarslan et al. prospectively evaluated a series of 30 Crowe type III–IV hips that had a normal ipsilateral knee. In the early period after THA, genu valgum deformity was observed in all knees, even when femoral shortening osteotomy was performed. They suggested that valgus deformity was an adaptive change resulting from tension along the iliotibial tract [3]. However, we found that limb lengthening and femoral shortening osteotomy were not influencing factors to knee valgus alignment. There were two possible reasons for the difference. On the one hand, different time points were used. In our study, patients may have adapted to the early change in knee after a minimum of 1-year rehabilitation exercise. One the other hand, the evaluation indicator was not exactly the same. We focused on an overall HKA angle from a full limb length standing AP radiographs, not Q angle as they measured.

Achievement of limb equalization was a technical challenge in THA for Crowe type IV hips, especially for the unilateral affected cases [16, 22]. LLD and pelvic obliquity were not uncommon among those patients. We hypothesized that longer limb and/or inadequately corrected pelvic obliquity that was towards ipsilateral side may force the knee into unphysiological biomechanics. And after a period of time, knee valgus deformity may develop due to medial laxity. However, only postoperative pelvic obliquity was a significant factor in our univariate analysis, and lost its significance in the adjusted model. Although the results did not meet expectations, we still believe that limb inequality and/or severe pelvic obliquity may contribute to knee valgus deformity. Joint line congruence angle of the knee was not measured in our study because we thought it can be masked by a weight-bearing radiograph [12]. Maybe a stress radiograph was more appropriate to assess the imbalance of mediolateral soft tissue.

Moreover, Kandemir et al. put forward a theoretical speculation that medialization of the hip center made the ipsilateral knee close to the contralateral knee. Those patients may try to walk with wider interfoot distance and therefore appeared genu valgum [4]. In clinical practice, we observed that in some patients with lower aTFA, the femur of the dislocated hip lay in a relatively adducent position to keep the lower leg vertical. After THA, the hip adduction was altered evidently, and hereupon the lower leg turned out. Kandemir et al. also suggested using a high-offset femoral component to compensate for the medialization of the hip center, further lowering the rate of knee valgus development [4]. However, although ΔFO was smaller in the valgus group, it was not a significant factor in our univariate analysis and there was no difference of the hip center medialization between two groups. Besides, high-offset stem cannot be conventionally used, because the choice was mainly dependent on the joint stability [23].

There are several limitations in this study. First, this is a retrospective study with its natural deficiency. However, the data we acquired were mainly from objective materials including radiographs and videos, with no recall bias. Second, our cohort is not a consecutive case series due to data integrity, and therefore selection bias may be not avoidable. Third, the sample size may be not large enough to identify all the influencing factors. Fourth, only one single femoral component was used in our cohort. However, the S-ROM modular stem is extensively used and adequate to adjust limb length, femoral anteversion, and femoral offset, which optimizes the outcome of hip reconstruction. Fifth, the duration of follow-up is variable. However, we suppose that a minimum of 1-year follow-up would warrant a constant knee alignment. Sixth, physical examination and clinical scoring were not performed for the knee joint. It would be more meaningful to conduct a prospective study to combine the clinical and radiographic data at sequential time points.

In conclusion, the postoperative mLDFA is a major factor related to knee valgus alignment after THA, which combines the preoperative anatomy and surgical reconstruction. Previously published factors including limb lengthening, restoration of femoral offset, LLD, and postoperative pelvic obliquity are not significant for knee valgus alignment.

## Data Availability

All data generated or analyzed during this study are included in this published article.

## Abbreviations

THA: Total hip arthroplasty
HKA: Hip–knee–ankle angle
mLDFA: Mechanical lateral distal femoral angle
AP: Anteroposterior
LLD: Limb length discrepancy
HHS: Harris Hip Score
DR: Digital radiography
mMPTA: Mechanical medial proximal tibial angle
aTFA: Anatomical tibiofemoral angle
aLDFA: Anatomical lateral distal femoral angle
NSA: Neck-shaft angle
HHC: Height of hip center
LHC: Lateral distance of hip center
FO: Femoral offset
ICC: Intraclass correlation coefficient
ORs: Odds ratios
CI: Confidence interval
